# The Association between Children’s and Parents’ Co-TV Viewing and Their Total Screen Time in Six European Countries: Cross-Sectional Data from the Feel4diabetes-Study

**DOI:** 10.3390/ijerph15112599

**Published:** 2018-11-21

**Authors:** Julie Latomme, Vicky Van Stappen, Greet Cardon, Philip J. Morgan, Mina Lateva, Nevena Chakarova, Jemina Kivelä, Jaana Lindström, Odysseas Androutsos, Esther M. González-Gil, Pilar De Miguel-Etayo, Anna Nánási, László R. Kolozsvári, Yannis Manios, Marieke De Craemer

**Affiliations:** 1 Department of Movement and Sports Sciences, Ghent University, 9000 Ghent, Belgium; vivstapp.vanstappen@ugent.be (V.V.S.); Greet.Cardon@UGent.be (G.C.); Marieke.DeCraemer@UGent.be (M.D.C.); 2 PRCPAN (Priority Research Centre for Physical Activity and Nutrition), School of Education, University of Newcastle, Callaghan NSW 2308, Australia; Philip.Morgan@newcastle.edu.au; 3 Clinic of Paediatric Endocrinology, Medical University Varna, 9002 Varna, Bulgaria; mina_pl@yahoo.com; 4 Clinical Center of Endocrinology, Medical University of Sofia, 1431 Sofia, Bulgaria; veni_chakarova@abv.bg; 5 National Institute for Health and Welfare, 00271 Helsinki, Finland; jemina.kivela@thl.fi (J.K.); jaana.lindstrom@thl.fi (J.L.); 6 Department of Nutrition and Dietetics, School of Health Science & Education, Harokopio University, 176 76 Athens, Greece; oandrou@hua.gr (O.A.); manios@hua.gr (Y.M.); 7 GENUD (Growth, Exercise, Nutrition and Development), University of Zaragoza, 50009 Zaragoza, Spain; esthergg@unizar.es (E.M.G.-G.); pilardm@unizar.es (P.D.M.-E.); 8 Department of Family and Occupational Medicine, University of Debrecen, Debreceni Egyetem (UoD), 4002 Debrecen, Hungary; nanasi.anna@sph.unideb.hu (A.N.); kolozsvari.laszlo@sph.unideb.hu (L.R.K.)

**Keywords:** screen time, co-TV viewing, childhood obesity, overweight, parents, children, sedentary behaviour

## Abstract

In many European children, high levels of screen time can be found, which is associated with several adverse health outcomes. Therefore, there is a need for identifying effective intervention strategies that reduce screen time in children. A factor that may contribute to excessive screen time in children may be “co-TV viewing” (i.e., the time that parents and children spend on watching TV together), as parents often recognize the importance of limiting children’s (individual) screen time, but often encourage TV viewing as a family because of its perceived benefits (e.g., educational purposes). The primary aim of this study was to investigate the (sex-specific) association between co-TV viewing and both children’s and parents’ screen time, and these associations were investigated across and within six European countries. In total, 10,969 parents (Mean_age_ = 40.7 ± 5.3 years, Mean_BMI_ = 24.4 ± 4.6) of primary school children (Mean_age_ = 8.2 ± 1.0 years, 49.0% boys, Mean_BMI_ = 17.3 ± 2.8) completed a questionnaire assessing co-TV viewing and screen time. Multilevel regression analyses were conducted. Across countries, positive associations were found between co-TV viewing and both children’s (β = 11.85, SE = 3.69, *p* < 0.001) and parents’ screen time (β = 14.47, SE = 4.43, *p* = 0.001). Similar associations were found in most (but not all) countries. The results suggest that targeting co-TV viewing might be a promising intervention strategy because of its potential to limit screen time of both children and parents.

## 1. Introduction

Screen time behaviours (and especially TV viewing and computer use) are the most common sedentary behaviours in school-aged children [1,2]. Recent evidence has shown that 19.0% to 31.7% of European primary school-aged children (6–9 years old) exceeds the internationally recommended guideline [3,4,5] of no more than 2 h recreational screen time per day on weekdays, and 57.4% to 71.2% exceeds this recommendation on weekend days [6]. Unhealthy patterns of sedentary behaviours, including screen time behaviours, are, however, associated with a number of negative physical and psychological health outcomes, including a higher risk of overweight/obesity, cardio-metabolic abnormalities, poor school performance, delayed language development, and psychological difficulties in school-aged children [7,8,9,10,11,12,13,14]. As these unhealthy patterns tend to track from childhood into adulthood [8,15], the establishment of healthy patterns of sedentary behaviours and especially screen time behaviours in children is of great importance [3,16]. Limiting these behaviours in children has therefore become an important focus in health promotion and childhood obesity prevention research [17,18]. 

However, recent systematic reviews investigating the effectiveness of lifestyle interventions targeting screen time behaviours in children have shown that intervention effects are often only small and/or short-termed [19,20]. Therefore, there is a need for interventions producing clinically meaningful and sustainable reductions in children’s screen time. An important step in developing such interventions is to identify effective intervention strategies. To do so, it is important to have a clear understanding of the factors related to children’s screen time behaviours. Modifiable factors can be the focus of future interventions, while non-modifiable factors, such as age and gender, can be used for identifying certain subgroups that are at increased risk. 

It is widely accepted that parents play a key role in establishing healthy behaviours in their children [21], including screen time behaviours, and research has shown that parental factors, such as parental support and involvement, increases the effectiveness of interventions targeting screen time in children [22,23]. This might suggest that interventions aiming to reduce screen time in children should include a focus on parental (social) factors and the (physical) home environment [24,25,26]. Parental (social) factors associated with children’s screen time include parents’ attitudes, parenting style (e.g., permissive, authoritative), parenting practices (e.g., rules and restrictions regarding screen time behaviours), and parents’ own screen time behaviours [27,28]. For example, it has been shown that parents’ screen time is positively associated with their children’s screen time [28,29]. Furthermore, parents’ perception of neighborhood safety and perceived distance to open and green spaces (e.g., parks) have been found to be associated with children’s screen time [30,31]. Physical environmental factors include, for example, the number of TV’s in the household, the access to TV’s and other electronic devices, and the presence of a TV in the child’s bedroom [29,32,33,34].

Another parental (social) factor related to children’s screen time is the time that parents and children spend on watching TV together (i.e., “co-TV viewing”). Until today, and to the best of our knowledge, this factor has only been investigated to a limited extent [35,36,37]. This could, however, be an important factor, because parents often understand the importance of limiting children’s (individual) screen time, but spend a lot of time watching TV together as a family [37,38]. Indeed, research has indicated that co-TV viewing is often seen as an important family activity, enhancing family functioning and facilitating social interaction and emotional connection, and is also used as an educational medium [39,40]. However, studies investigating this factor showed a significant positive association between co-TV viewing and children’s screen time [35,36,37], meaning that when parents and children spent more time on co-TV viewing, children’s screen time was higher. This suggests that co-TV viewing could be a potential focus of interventions aiming to limit screen time in children. However, before meaningful conclusions or recommendations for future research can be made on this, a next important step is to investigate the association between co-TV viewing and parents’ screen time too. This is because parents act as important role models for their children and set the overall context of screen time behaviour in the home environment [41,42]. If an association between co-TV viewing and parents’ screen time would be found, this would imply that focusing on co-TV viewing could be a promising intervention strategy as it could lead to reductions in children’s and parents’ screen time simultaneously. 

In addition to investigating the association between co-TV viewing and both parents’ and children’s screen time, it could also be useful to look at the influence of an individual’s biological sex in these associations, which has not been investigated previously. Looking at sex-specific associations may be useful for identifying subgroups that require special attention in intervention studies. Sex-specific associations have already been found in previous studies on co-TV viewing and screen time in children. For example, Salmon et al. (2015) found an association only for boys between family TV viewing and exceeding screen time recommendations in boys [37]. Furthermore, a study by Totland et al. (2013) found only a positive association between mothers’ PC/video game time and their sons’ PC/video game time, and between fathers’ PC/video game time and their daughters’ PC/video game time [43].

Last, a major shortcoming in the literature is that existing evidence on sedentary behaviours, including screen time behaviours, predominantly comes from research conducted in high-income countries [44]. Given the different occupational and socio-cultural structures, environmental factors (e.g., safety, climate), etc. in middle- to low-income countries, more evidence derived from research in these countries is needed [45]. The current study addresses this shortcoming by investigating data from six European countries, representing different socio-economic levels. Another added value of studying large-scale international data is that it allows an examination of associations regardless of the specific characteristics of a country, and, at the same time, it allows comparison between countries in order to examine the role of a certain factor (co-TV viewing in this case) in different countries. 

Taken together, the aim of the present study is to investigate associations, across and within six European countries, between co-TV viewing and both children’s and parents’ screen time, and determine whether these associations are sex-specific. With this, we aim to inform and guide future research and intervention studies in identifying new, more effective strategies for limiting screen time in children to prevent childhood obesity.

## 2. Materials and Methods 

### 2.1. Study Background

This study used cross-sectional data from the “Feel4Diabetes-study”, which was conducted in six European countries representing low income countries (Bulgaria and Hungary), high income countries (Belgium and Finland), and countries under austerity measures (Greece and Spain). The Feel4Diabetes-study is registered within the clinical trials registry clinical_trials.gov, ID: 643708. More details on this study and its design can be found elsewhere (www.feel4diabetes-study.eu) [46]. 

### 2.2. Measures

Within the Feel4Diabetes-study, a questionnaire was developed to be completed (at home) by one of the parents/primary caregivers. For the present study, only relevant socio-demographics (i.e., children’s and parents’ age and sex) and relevant lifestyle behaviour measures (i.e., measures of (co-) screen time and physical activity (PA)) were used. Children’s and parents’ screen time was assessed in two questions, i.e., “How many hours per day do you/does your child usually spend on screen activities, such as TV/DVD viewing, computer/smartphone/tablet use, and video games (activities at work/school not included) on (a) weekdays, and on (b) weekend days?”. Answer options varied on a 10-point scale, ranging from “none” to “7 or more hours/day”, with a one hour range in other options, e.g., “2 to less than 3 h/day”. These categorical variables were recoded into numerical variables using the midpoint method (e.g., “2 to less than 3 h/day” was recoded into 150 min/day, “3 to less than 4 h/day was recoded into 210 min/day, “7 or more hours/day” was recoded into 450 min/day, etc.) [47], and the average daily amount of children’s and parents’ screen time (min/day) was then calculated using the following formula: (screen time_weekdays_ × 5 + screen time_weekenddays_ × 2)/7. Co-TV viewing of children and parents was assessed with the question “How often do you watch TV together with your child?”. Answer options varied on a 5-point Likert scale with options “Never”, “Rarely”, “Sometimes”, “Often”, and “Very often”. The test-retest reliability of children’s and parents’ (co-) screen time measures were ranked as ‘good’ to ‘excellent’ (ICC range = 0.65 to 0.83), except for parents’ screen time on weekdays, which was ranked as ‘poor’ (ICC = 0.38). 

Parents’ Body Mass Index (BMI, in kg/m²) was calculated based on their self-reported weight and height, and children’s BMI was calculated based on their objective weight and height. More specifically, children were measured at schools by a team of researchers between April and June 2016. Height was measured using the Seca 2017 stadiometer for mobile height measurement, and weight was measured using the Seca 813 digital flat scale. Children’s and parents’ PA levels were assessed in two questions of the questionnaire, i.e., “In the previous week, how many days were you/was your child active for at least 30 min/day (parent), 60 min/day (child) (a) on weekdays, and (b) on weekend days? With ‘active’ we mean any kind of movement that makes you sweat a little and increases your heart rate, for example, cycling, dancing, gardening, fitness, etc.”. For weekdays, possible answer options varied on a 6-point scale ranging from “none” to “5 days”. For weekend days, possible answer options varied on a 3-point scale ranging from “none” to “2 days”. These categorical values were then recoded into numerical variables (i.e., none was recoded into 0, 1 day was recoded into 1, etc.). The sum of these two variables was used in the analyses as a measure of the children’s and parents’ amount of PA, reflecting the number of days parents/children reached the PA guideline (i.e., 30 min/day for parents, 60 min/day for children).

### 2.3. Data Analysis

**Figure 1:**
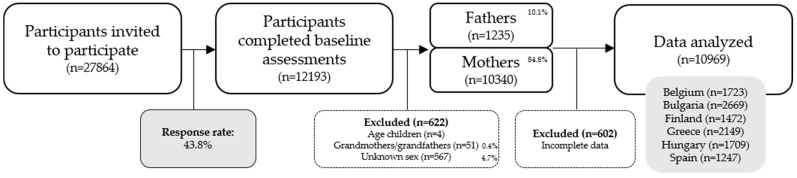
Flow diagram of participants throughout the study.

Only data reported by parents/caregivers of primary school aged children aged between 5 and 13 years old were included in the present study (*n* = 622 were excluded for not meeting these inclusion criteria), see Figure 1 for a more detailed description. All participants who had incomplete data on the outcome variables or had no data on co-TV viewing were also excluded from the dataset (*n* = 602). In total, data for 10,969 participants were included in the analyses. Descriptive statistics were computed to describe the sample characteristics, using IBM SPSS Statistics for Windows, version 25.0 (SPSS Inc., Chicago, IL, USA) [48]. To investigate differences between countries in co-TV viewing and total screen time of children and parents, a one-way ANOVA was conducted. The hierarchical structure of the data, with children being nested within classes nested within schools nested within countries, and the adequate sample size for conducting multilevel analyses [49], allowed us to employ multilevel regression analyses to examine the associations between children’s and parents’ co-TV viewing and their total screen time, and to look whether these associations differed by parent and/or child sex. To investigate the associations across the six European countries (i.e., in the total sample), multilevel analyses were conducted using MLwiN 3.00 (http://www.bristol.ac.uk/cmm/software/mlwin/) [50] with four levels: Child, class, school, and country. First, a null model or intercept-only model including only the outcome variable was estimated for children’s screen time and parents’ screen time, respectively. Second, children’s and parents’ age, sex (0 = boy/man, 1 = girl/woman), BMI, and PA levels were inserted in the model as covariates to control for their potential confounding effect. Last, co-TV viewing was included as a (continuous) predictor. In the same model, also the two-way interaction effects, Co-TV viewing × Sex_child_ and Co-TV viewing × Sex_parent_, and the three-way interaction effect, Co-TV viewing × Sex_child_ × Sex_parent_, were added. Only this model (i.e., full model) will be reported in the results for children’s and parents’ screen time. Before investigating country-specific associations, we first checked whether the explained variance in the outcome variables was significant at the country-level in the null model for the total sample. When this was the case, the multilevel analyses were repeated for each country separately with three levels: Child, class, and school. For each outcome variable, the main effect of Co-TV viewing, the two-way interaction effects of Co-TV viewing × Sex_child_ and Co-TV viewing × Sex_parent_, and the three-way interaction effect of Co-TV viewing × Sex_child_ × Sex_parent_ were considered. Statistical significance level was set on *p* < 0.05.

### 2.4. Ethics Approval and Consent to Participate

All applicable institutional regulations pertaining to the ethical use of human volunteers were followed during this research. Ethical approval was provided by the Ethical Committees of all participating European countries (i.e., Ethical committee of Ghent University Hospital (Belgium), Committee for the Ethics of the Scientific Studies (KENI) at the Medical University of Varna and the Municipality of Sofia (Bulgaria), Ethics Committee of Harokopio University of Athens, the Greek Ministry of Education, Research and Religious Affairs and the Municipalities of Kallithea, Peristeri, Piraeus and Keratsini-Drapetsona (Greece), CEICA (Comité Etico de Investigacion Clinica de Aragon (Spain), Ethics Committee of THL (Finland), and the Bioethics Committee of University of Debrecen (Hungary). Participants received an information letter in which they were briefly informed about the purpose of the study and signed a written informed consent.

## 3. Results

### 3.1. Descriptives

In total, data for 10,969 parents/caregivers (Mean_age_ *=* 40.7 ± 5.3 years, 89.2% mothers) of primary school aged children (Mean_age_ *=* 8.3 ± 1.0 years, 49.0% boys) were analyzed. The flow diagram of participants throughout the study can be found in Figure 1. Descriptive statistics of the sample and variables can be found in Table 1. 

**Figure 2:**
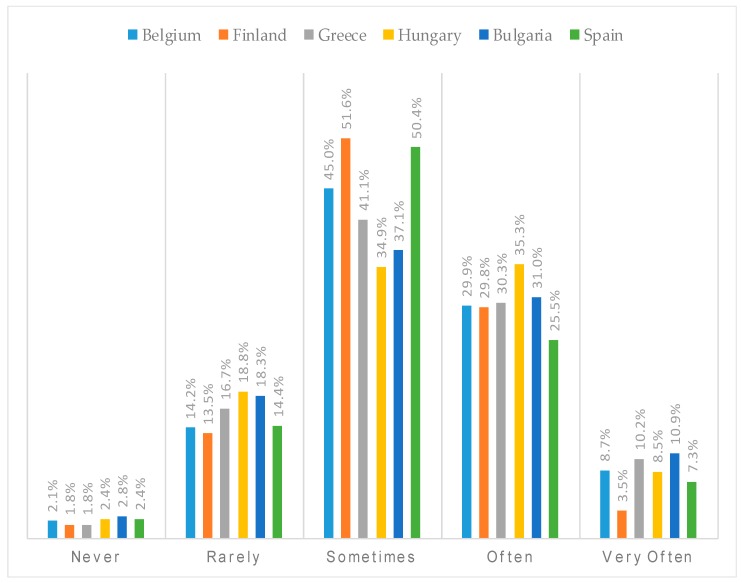
Distribution of the percentages across the categories of co-TV viewing for each of the six European countries.

**Figure 3:**
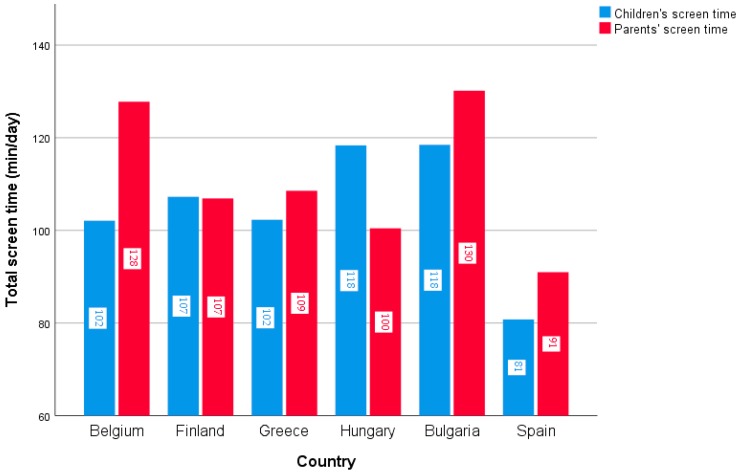
Average amount of children’s (in blue) and parents’ (in red) total screen time for each of the six European countries.

In the total sample, 2.2% of the parents and children never co-watched TV, 42.1% co-watched TV sometimes, 30.6% often, and 8.6% very often (see Table 1). Country-specific percentages can be found in Figure 2, which differed significantly between countries (*χ*^2^ = 221.1, *p* < 0.001). Children’s and parents’ total screen time was on average 106.9 min/day and 113.3 min/day in the total sample, respectively, with differences found between countries for both children’s and parents’ total screen time (F = 68.9, *p* < 0.001 and F = 67.6, *p* < 0.001, respectively). Country-specific data for both children’s and parents’ screen time is represented in Figure 3. For children’s screen time, post-hoc tests revealed that children’s screen time was the lowest in Spain (80.8 min/day), which differed significantly from Belgium (102.2 min/day), Greece (102.31 min/day), and Finland (107.3 min/day), which again differed significantly from Hungary (118.2 min/day) and Bulgaria (118.4 min/day), who had the highest levels of children’s screen time. For parents’ screen time, post-hoc tests revealed that Spain and Hungary had the lowest levels of parents’ screen time (91.3 min/day and 100.0 min/day, respectively), followed by Finland (106.8 min/day), Greece (108.4 min/day), and Belgium (127.9 min/day), and, last, Bulgaria (130.2 min/day), where parents’ screen time was the highest.

### 3.2. Association of Co-TV Viewing and Screen Time of the Child

#### 3.2.1. Total Sample

**Figure 4:**
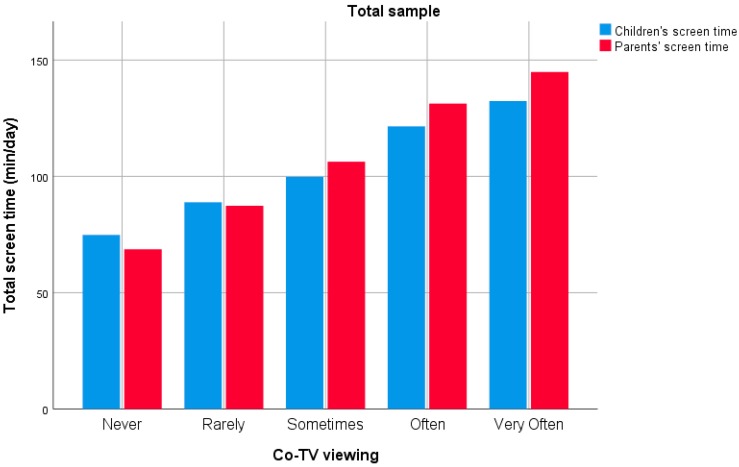
Graphical representation of the association between co-TV viewing and children’s (in blue) and parents’ (in red) screen time (min/day) for the total sample.

Results for the association between co-TV viewing and children’s screen time are shown in Table 2, and graphically represented in Figure 4. In the total sample, co-TV viewing was significantly and positively associated with children’s screen time (β = 11.85, SE = 3.69, *p* < 0.001). The more parents and children watched TV together, the higher children’s total screen time was (i.e., a one-category change in co-TV viewing was associated with an increase of 11.85 min/day in children’s screen time). No significant two-way interaction effects were found in the total sample for Co-TV viewing × Sex_child_ or Co-TV viewing × Sex_parent_, nor a three-way interaction effect of Co-TV viewing × Sex_child_ × Sex_parent_ (all *p* > 0.05). 

#### 3.2.2. Effects per Country

**Figure 5:**
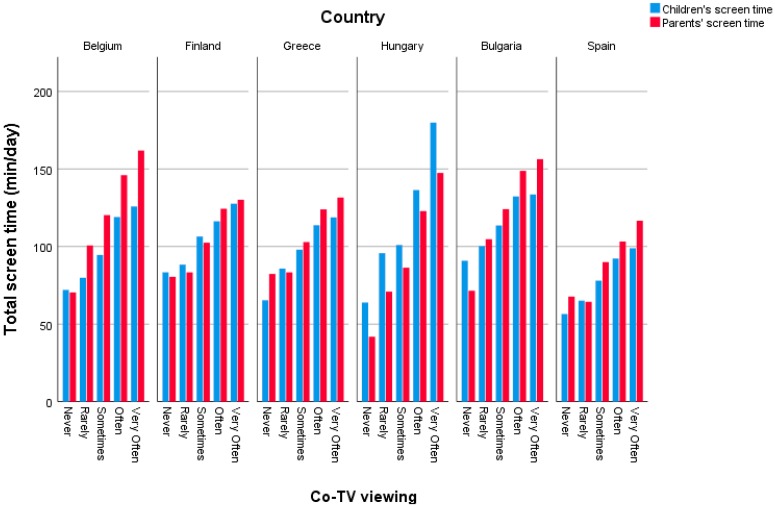
Graphical representation of the association between co-TV viewing and children’s (in blue) and parents’ (in red) screen time (min/day) for the six European countries.

The random part of the null model for children’s screen time showed that the variance at country-level differed significantly from zero (13.1%, χ^2^(1) = 25.17, *p* < 0.001), meaning that investigating country-specific associations was appropriate for children’s screen time. Country-specific results for children’s screen time are shown in Table 2, and graphically represented in Figure 5. In Belgium, Hungary, and Spain, co-TV viewing was significantly and positively associated with children’s screen time (Belgium: β = 19.51, SE = 5.98, *p* = 0.001; Hungary: β = 28.46, SE = 9.34, *p* = 0.002; Spain: β = 25.90, SE = 8.24, *p* = 0.002), meaning that in these countries, a one-category change in co-TV viewing was associated with an increase in children’s screen time of 19.51 min/day in Belgium, 28.46 min/day in Hungary, and 25.90 min/day in Spain. For Finland and Bulgaria, no association between co-TV viewing and children’s screen time was found. For Greece, the two-way interaction effect of Co-TV viewing × Sex_parent_ was found to be significant (β = 20.29, SE = 6.70, *p* = 0.002), meaning that the effect of co-TV viewing on children’s screen time differed according to the sex of the parent with who the child co-watched TV. Further stratified analyses (i.e., conducting the analysis separately for the mother-child dyads and father-child dyads) showed that co-TV viewing was only significantly and positively associated with children’s screen time for the mother-child dyads (β = 14.19, SE = 2.40, *p* < 0.001); a one-category increase in mother and child co-watching TV was associated with an increase of 14.19 min/day in children’s screen time. For Hungary, the three-way interaction effect of Co-TV viewing × Sex_parent_ × Sex_child_ was found to be significant (β = 28.46, SE = 9.34, *p* = 0.002). Further stratified analyses showed that co-TV viewing was significantly and positively associated with children’s screen time for the son-father dyads (β = 33.09, SE = 10.75, *p* = 0.002), son-mother dyads (β = 18.18, SE = 3.17, *p* < 0.001), and daughter-mother dyads (β = 22.94, SE = 2.94, *p* < 0.001), but not for the daughter-father dyads (*p* > 0.05). A one-category increase in son and father co-TV viewing was associated with an increase of 33.09 min/day in boys’ screen time, a one-category increase in son and mother co-TV viewing was associated with an increase of 18.18 min/day in boys’ screen time, and a one-category increase in daughter and mother co-TV viewing was associated with an increase of 22.94 min/day in girls’ screen time. No other significant main or interaction effects were found (all *p* > 0.05).

### 3.3. Association of Co-TV Viewing and Screen Time of the Parent

#### 3.3.1. Total Sample

Results for the association between co-TV viewing and parents’ screen time are shown in Table 3, and graphically represented in Figure 4. In the total sample, co-TV viewing was significantly and positively associated with parents’ screen time (β = 14.47, SE = 4.43, *p* = 0.001). The more parents and children watched TV together, the higher parents’ total screen time was (i.e., a one-category increase in co-TV viewing was associated with an increase of 14.47 min/day in parents’ screen time). No significant two-way interaction effects were found in the total sample for Co-TV viewing × Sex_child_ or Co-TV viewing × Sex_parent_, nor a three-way interaction effect of Co-TV viewing × Sex_child_ × Sex_parent_ (all *p* > 0.05).

#### 3.3.2. Effects per Country

The random part of the null model for parents’ screen time showed that the variance at the country-level differed significantly from zero (12.0%, χ^2^(1) = 24.84, *p* < 0.001), meaning that investigating country-specific associations was appropriate for parents’ screen time. Country-specific results for parents’ screen time are shown in Table 3, and graphically represented in Figure 5. Only in Belgium, Finland, and Hungary, co-TV viewing was significantly and positively associated with parents’ screen time (β = 44.20, SE = 9.46, *p* < 0.001). A one-category change in co-TV viewing was associated with an increase in parents’ screen time of 16.33 min/day in Belgium, 20.11 min/day in Finland, and 39.18 min/day in Hungary. For Greece, the effect of co-TV viewing on parents’ screen time differed according to the sex of the parent, as a significant two-way interaction effect of Co-TV viewing × Sex_parent_ was found (β = −23.31, SE = 8.76, *p* = 0.008). Further stratified analyses (i.e., conducting the analyses separately for the mother-child dyads and the father-child dyads) showed that co-TV viewing was only significantly and positively associated with parents’ screen time for the mother-child dyads (β = 18.89, SE = 3.12, *p* < 0.001); a one-category increase in mother and child co-TV viewing was associated with an increase of 18.89 min/day in mothers’ screen time. For Hungary, both the two-way interaction effect of Co-TV viewing × Sex_child_ (β = −36.66, SE = 12.19, *p* = 0.003) and the three-way interaction effect of Co-TV viewing × Sex_parent_ × Sex_child_ (β = 37.19, SE = 12.88, *p* = 0.004) were significant, meaning that the effect of co-TV viewing on parents’ screen time differed according to the sex of the child and the sex of the parent. Further stratified analyses showed that co-TV viewing was significantly and positively associated with parents’ screen time for the father-son dyads (β = 40.44, SE = 9.66, *p* < 0.001), mother-son dyads (β = 28.72, SE = 3.11, *p* < 0.001), and mother-daughter dyads (β = 27.20, SE = 2.87, *p* < 0.001), but not the father-daughter dyads (*p* > 0.05). A one-category increase in father and son co-TV viewing was associated with an increase of 40.44 min/day in fathers’ screen time, a one-category increase in mother and son co-TV viewing was associated with an increase of 28.72 min/day in mothers’ screen time, and a one-category increase in mother and daughter co-TV viewing was associated with an increase of 27.20 min/day in mothers’ screen time. No other significant main or interaction effects were found (all *p* > 0.05).

## 4. Discussion

The present study investigated the (sex-specific) associations between co-TV viewing and both children’s and parents’ screen time across and within six European countries, aiming to inform and guide future research in identifying effective intervention strategies to limit screen time in children.

In the total sample, relatively high levels of co-TV viewing were found. Only 2.2% of the parents and children never co-watched TV and 39.2% co-watched TV often to very often. These figures show that co-TV viewing is a common family behaviour, which has also been stated in other studies [51,52]. This is, however, worrying because research has shown that TV viewing is often seen as an important family activity that, in contrast to individual screen time, should not be limited [51]. Even more, TV viewing as a family is often encouraged by parents because of its perceived benefits, such as parental involvement, promoting social interaction, emotional connectedness, and its educational purposes [39,40,53]. Furthermore, our results showed across European countries a positive association between co-TV viewing and both children’s and parents’ screen time, which was not sex-specific. The positive association between co-TV viewing and children’s screen time was also found in previous studies investigating this association [35,36,37]. However, the association between co-TV viewing and parents’ screen time has not been investigated yet, thus contributing to the existing literature.

Our findings suggest that targeting co-TV viewing may indeed be a novel and effective intervention strategy for limiting screen time in children. This is because research has shown that screen-time behaviour is a good proxy of sedentary behaviour and often the easiest sedentary behaviour to target in interventions, while, on the other hand, also having potential to limit children’s and parents’ screen time at the same time. Also, targeting parents is important, because parents act as important role models and set the general context regarding screen time in the household. Previous studies already have shown the relevance of simultaneously targeting parents and children in the context of PA and nutrition [54,55]. These studies showed that when fathers and children were motivated to engage in co-PA and encouraged each other in healthy eating, both fathers’ and children’s health (e.g., weight status) and health behaviors (e.g., PA levels) significantly improved. These effects could be explained by the reciprocal reinforcement of adopting healthier behaviors and the additional motivation for parents to model healthy behaviors and create a healthy environment for their children [55].

As co-TV viewing is a common family behaviour during childhood, but remains an important family activity when children grow older [53,56], it may be important to target co-TV viewing already from childhood so that healthy (family) habits are established already from an early age. Interventions targeting co-TV viewing could, for example, change parents′ perception and attitude towards co-TV viewing and highlight the detrimental effects of excessive co-TV viewing, as well as the importance of limiting this behaviour. Furthermore, information can be given on how to incorporate the perceived benefits of co-TV viewing into new contexts, for example, by providing parents and children alternative activities that can be done as a family (i.e., information on how they can replace co-TV viewing with other fun or educational family activities), or they can be encouraged to devise and implement alternatives themselves [56]. 

Country-specific analyses revealed a positive association between co-TV viewing and children’s screen time for Spain, between co-TV viewing and parents’ screen time for Finland, and between co-TV viewing and both children’s and parents’ screen time for Belgium, Greece, and Hungary. For Greece and Hungary these associations were sex-specific; for Greece, only a positive association was found between mother-child co-TV viewing and their screen time, which means that the more mothers watched TV together with their child, the higher their total screen time was. No other sex-specific associations were found for Greece. For Hungary, all sex-specific associations were significant (i.e., mother-son, mother-daughter, and father-son), except the association between father-daughter co-TV viewing and their screen time. Reasons for the presence or absence of a certain association in a certain country, or reasons for the sex-specific associations found in Greece and Hungary, are currently still unclear so more in-depth quantitative and qualitative research investigating this is needed.

As a positive association was found between co-TV viewing and both children’s and parents’ in the total sample across European countries (i.e., independent of country-specific characteristics), this suggests that targeting co-TV viewing can be an international, cross-European intervention strategy to limit screen time in children, benefiting families (parents and children) from different countries with different socio-economic levels. However, because associations differed between countries, we can suggest that co-TV viewing can be used as an international intervention strategy if there is room for small, local adaptations or if different emphases can be made per country.

The present study also has some limitations. First, we only considered the total amount of screen time of children and parents, with no distinction being made between different types/subdomains of screen time (e.g., computer use, TV viewing, smartphone use, etc.). It could, however, be useful to investigate associations between co-TV viewing and the different subdomains of screen time too. Moreover, in the present study, the amount of time spent on co-TV viewing (partially) overlaps with total screen time. To address this, co-TV viewing could be measured in more detail, for example, by including quantitative information on frequency (e.g., times/week) and duration (e.g., minutes/day). Further, also qualitative data on co-TV viewing could be included to gain more insight into, for example, the reasons and individual- and family-related benefits of co-TV viewing. Finally, in addition to examining parent-child co-TV viewing, also co-screen time between siblings and its influence on total screen time of children can be further explored in future studies. Another limitation is that no conclusions about causality can be drawn using cross-sectional data, and a longitudinal design is recommended to be able to do so. As self-reported data were used, the possibility of social desirable responses is also a limitation. Children’s data were based on parental report, which is a subjective proxy-measure that also can be biased. Furthermore, although 80% of the questions had a test-retest reliability ranked as good to excellent, the test-retest reliability of one of the variables could be improved (i.e., the question on parents’ screen time on weekdays). Despite these shortcomings that are inherent to questionnaires, they are common and often the only possible method in studies measuring screen time behaviours [56,57]. Objective measures of co-TV viewing and objectively measured sedentary behaviour may overcome this issue and are therefore recommended for future research. For example, posture monitors (e.g., inclinometers/Activpals) could be used in combination with time-use diaries for future research. Strengths of the present study are that data were included from six European countries, providing a large sample (*n* = 10,969) and increasing the generalizability of the results. 

## 5. Conclusions

The present study found that higher levels of co-TV viewing were associated with higher levels of both children’s and parents’ total screen time in six European countries. Targeting co-TV viewing might therefore be a novel and promising intervention strategy to limit screen time in children, as it has the potential to target both children and their parents, who function as important role models and set the overall context for healthy behaviours, including screen time behaviours in the household. As associations were found across European countries with only small country-specific differences, targeting co-TV viewing could be a cross-European intervention strategy if there is room for small, local adaptations and if different accents can be laid per country. More in-depth research on co-TV viewing (e.g., quantifying co-TV viewing, including qualitative data) is, however, recommended for future research. 

## Figures and Tables

**Table 1:** Descriptive statistics.

Sample Characteristics	Parents			Children		
	All Parents	Fathers	Mothers	All Children	Boys	Girls
***n***	10,969	1183	9786	10,696	5380	5589
**Age, in years (SD)**	40.7 (5.3)	43.6 (5.9)	40.3 (5.1)	8.2 (1.0)	8.2 (1.0)	8.2 (1.0)
**Sex, %**	100	10.8	89.2	100	49.0	51.0
**BMI ^a^, in kg/m² (SD)**	24.4 (4.6)	27.1 (3.9)	24.0 (4.6)	17.3 (2.8)	17.3 (2.8)	17.3 (2.9)
**Screen time, in min/day (SD)**	113.3 (80.7)	132.2 (84.0)	111.0 (80.0)	106.9 (67.0)	111.8 (67.8)	102.3 (65.9)
**Co-TV viewing, % (range)**	Parent-child dyad	Father-child dyad	Mother-child dyad		Son-parent dyad	Daughter-parent dyad
Never	2.2 (1.8–2.8) ^b^	2.0 (1.3–3.8)	2.3 (1.8–2.5)	(see parent-child dyad)	1.9 (1.4–2.6)	2.6 (1.9–3.0)
Rarely	16.3 (13.5–18.8) ^b^	14.0 (10.3–20.5)	16.6 (13.9–18.7)	(see parent-child dyad)	16.0 (11.2–18.8)	16.6 (14.1–18.8)
Sometimes	42.1 (34.9–51.6) ^b^	40.5 (24.6–53.7)	42.4 (36.1–51.3)	(see parent-child dyad)	42.9 (33.7–54.0)	41.6 (36.0–49.2)
Often	30.6 (25.5–35.5) ^b^	34.6 (30.3–43.9)	30.1 (24.3–34.3)	(see parent-child dyad)	30.8 (25.9–36.9)	30.3 (25.1–33.8)
Very often	8.6 (3.5–10.9) ^b^	8.9 (4.0–12.4)	8.6 (3.4–11.0)	(see parent-child dyad)	8.4 (3.4–10.4)	8.9 (3.5–11.8)

This table provides means (SD) for the continuous variables and frequency (%) for the categorical variables. ^a^ Body Mass Index. b These numbers are identical for the children as it concerns parents and children co-TV viewing.

**Table 2:** Screen time of the child. Results of multilevel model analyses on children′s screen time (min/day) (full model).

	Total (*n* = 9815)	Belgium (*n* = 1581)	Bulgaria (*n* = 2421)	Finland (*n* = 1168)	Greece (*n* = 1929)	Hungary (*n* = 1564)	Spain (*n* = 1152)
Fixed Part	β (SE)	β (SE)	β (SE)	β (SE)	β (SE)	β (SE)	β (SE)
Intercept	117.97 (3.31)	114.65 (4.47)	121.14 (6.50)	126.26 (4.66)	119.32 (4.66)	140.53 (10.48)	89.38 (4.95)
BMI child	1.21 (0.24) ***	2.30 (0.74) **	1.42 (0.47) **	ns	ns	ns	2.15 (0.64) **
BMI parent	0.33 (0.15) *	ns	ns	ns	0.79 (0.32) *	ns	ns
Age child	5.79 (0.71) ***	5.70 (1.69) **	8.82 (1.58) ***	10.60 (1.64) ***	ns	ns	ns
Age parent	−0.40 (0.13) **	ns	ns	ns	ns	ns	ns
Sex child ^a^	−10.37 (3.79) **	−8.17 (2.95) **	−9.33 (2.60) ***	−18.86 (3.01) ***	−11.98 (2.92) ***	ns	ns
Sex parent ^b^	−8.78 (2.16) ***	−9.21 (4.46) *	ns	−10.85 (4.67) *	−12.72 (4.61) **	ns	ns
PA level child	−3.33 (0.44) ***	ns	−4.05 (0.86) ***	−8.91 (1.24) ***	−3.32 (0.94) ***	ns	ns
PA level parent	2.25 (0.33) ***	ns	1.87 (0.68) **	ns	ns	3.99 (0.98) ***	ns
**Co-TV viewing**	11.85 (3.69) ***	19.51 (5.98) **	ns	ns	ns	28.46 (9.34) **	25.90 (8.24) **
Co-TV viewing * Sex child	ns	ns	ns	ns	ns	ns	ns
Co-TV viewing * Sex parent	ns	ns	ns	ns	20.29 (6.70) **	ns	ns
Stratified analysis ^c^							
Father-child dyads (*n* = 250)	-	-	-	-	ns	-	-
Mother-child dyads (*n* = 1679)	-	-	-	-	14.19 (2.40) ***	-	-
Co-TV viewing * Sex child * Sex parent	ns	ns	ns	ns	ns	26.00 (12.72) *	ns
Stratified analysis ^c^							
Son-father dyads (*n* = 84)	-	-	-	-	-	33.09 (10.75) **	-
Son-mother dyads (*n* = 667)	-	-	-	-	-	18.18 (3.17) ***	
Daughter-father dyads (*n* = 74)	-	-	-	-	-	ns	-
Daughter-mother dyads (*n* = 739)	-	-	-	-	-	22.94 (2.94) ***	-
**Random part**	**σ^2^ (SE)**	**σ^2^ (SE)**	**σ^2^ (SE)**	**σ^2^ (SE)**	**σ^2^ (SE)**	**σ^2^ (SE)**	**σ^2^ (SE)**
Country-level variance	160.01 (37.28) ***	-	-	-	-	-	-
School-level variance	0.00 (0.00) ^d^	64.07 (34.96)	179.98 (72.77) *	16.99 (20.07)	32.00 (32.30)	927.95 (390.41) *	53.07 (36.07)
Class-level variance	523.47 (214.20) *	0.00 (0.00) ^d^	34.64 (40.45)	0.00 (0.00) ^d^	81.16 (61.81)	192.93 (80.44) *	82.68 (57.01)
Individual-level variance	3421.80 (217.64) ***	3314.11 (119.81) ***	3989.45 (120.05) ***	2572.06 (107.45) ***	3902.99 (134.92) ***	5054.01 (187.23) ***	2382.68 (108.62) ***

* *p* < 0.05, ** *p* < 0.01, *** *p* < 0.001. All analyses were adjusted for children’s and parents’ age, sex, BMI, and PA levels. Reference category = 0; ^a^ 0 = boy, 1 = girl; ^b^ 0 = man, 1 = woman. ^c^ For the stratified analyses, β (SE) of the variable of co-TV viewing is given; ^d^ an estimated variance of zero means that this level does not help explain any of the overall variability present in the data; ns = not significant.

**Table 3:** Screen time of the parent. Results of multilevel model analyses on parents’ screen time (min/day) (full model).

	Total (*n* = 9830)	Belgium (*n* = 1588)	Bulgaria (*n* = 2420)	Finland (*n* = 1165)	Greece (*n* = 1931)	Hungary (*n* = 1568)	Spain (*n* = 1158)
Fixed Part	β (SE)	β (SE)	β (SE)	β (SE)	β (SE)	β (SE)	β (SE)
Intercept	130.35 (3.92)	139.14 (5.64)	145.12 (7.64)	124.11 (5.01)	139.82 (6.06)	125.40 (6.79)	107.45 (6.51)
BMI child	−0.64 (0.29) *	ns	ns	ns	ns	ns	ns
BMI parent	1.28 (0.18) ***	1.40 (0.50) **	1.06 (0.41) *	1.85 (0.38) ***	2.10 (0.42) ***	ns	1.49 (0.49) **
Age child	ns	ns	ns	ns	ns	ns	ns
Age parent	−0.94 (0.16) ***	ns	ns	−1.30 (0.33) ***	−0.80 (0.38) *	ns	2.33 (2.28) ***
Sex child ^a^	ns	ns	ns	ns	ns	ns	ns
Sex parent ^b^	−22.11 (2.62) ***	−12.32 (5.82) *	ns	−20.21 (5.13) ***	−35.48 (6.02) ***	−27.36 (6.43) ***	−21.40 (6.61) **
PA level child	ns	ns	ns	−3.86 (1.37) **	ns	ns	4.98 (1.40) ***
PA level parent	−0.97 (0.40) *	ns	ns	−2.45 (0.96) *	−1.98 (0.89) *	ns	ns
**Co-TV viewing**	14.47 (4.43) **	16.33 (7.81) *	ns	20.11 (8.41) *	ns	39.18 (9.38) ***	ns
Co-TV viewing * Sex parent	ns	ns	ns	ns	23.31 (8.76) **	ns	ns
Stratified analysis ^c^							
Father-child dyads (n = 250)	-	-	-	-	ns	-	-
Mother-child dyads (n = 1681)	-	-	-	-	18.89 (3.12) ***	-	-
Co-TV viewing * Sex child	ns	ns	ns	ns	ns	-36.66 (12.19) **	ns
Stratified analysis ^c^							
Parent-son dyads (n = 753)	-	-	-	-	-	40.44 (9.66) ***	-
Parent-daughter dyads (n = 815)	-	-	-	-	-	ns	-
Co-TV viewing * Sex parent * Sex child	ns	ns	ns	ns	ns	37.19 (12.88) **	ns
Stratified analysis ^c^							
Father – son dyads (n = 84)	-	-	-	-	-	40.44 (9.66) ***	-
Father – daughter dyads (n = 73)	-	-	-	-	-	ns	-
Mother – son dyads (n = 669)	-	-	-	-	-	28.72 (3.11) ***	-
Mother – daughter dyads (n = 742)	-	-	-	-	-	27.20 (2.87) ***	-
**Random part**	**σ^2^ (SE)**	**σ^2^ (SE)**	**σ^2^ (SE)**	**σ^2^ (SE)**	**σ^2^ (SE)**	**σ^2^ (SE)**	**σ^2^ (SE)**
Country-level variance	214.40 (51.03) ***		-	-	-	-	-
School-level variance	0.00 (0.00) ^d^	4.17 (34.71)	42.57 (35.21)	0.00 (0.00) ^d^	51.20 (52.11)	59.59 (49.03)	0.00 (0.00) ^d^
Class-level variance	846.67 (315.13) **	0.00 (0.00) ^d^	94.51 (73.52)	0.00 (0.00) ^d^	79.18 (99.87)	119.88 (69.54)	0.00 (0.00) ^d^
Individual-level variance	4990.77 (319.73) ***	5730.31 (206.16) ***	6915.43 (208.17) ***	3115.73 (129.10) ***	6721.43 (231.96) ***	5152.52 (190.49) ***	4705.76 (195.57) ***

*p* < 0.05, ** *p* < 0.01, *** *p* < 0.001. All analyses were adjusted for children’s and parents’ age, sex, BMI, and PA levels. Reference category = 0; ^a^ 0 = boy, 1 = girl; ^b^ 0 = man, 1 = woman. ^c^ For the stratified analyses, β (SE) of the variable of co-TV viewing is given. ^d^ an estimated variance of zero means that this level does not help explain any of the overall variability present in the data.

## The Feel4Diabetes-study group consists of

(1) Coordinator: Yannis Manios, (2) Project manager: Odysseas Androutsos, (3) Steering Committee: Yannis Manios, Greet Cardon, Jaana Lindström, Peter Schwarz, Konstantinos Makrilakis, Lieven Annemans, Dimitrios Kakoulis.

## The Feel4Diabetes member group

Yannis Manios, Meropi Kontogianni, Odysseas Androutsos, George Moschonis, Konstantina Tsoutsoulopoulou, Christina Mavrogianni, Christina Katsarou, Eva Karaglani, Eirini Efstathopoulou, Ioanna Kechribari, Konstantina Maragkopoulou, Effie Argyri, Athanasios Douligeris, Mary Nikolaou, Eleni-Anna Vampouli, Katerina Kouroupaki, Roula Koutsi, Elina Tzormpatzaki, Eirini Manou, Panagiota Mpinou, Alexandra Karachaliou, Christina Filippou, Amalia Filippou, Jaana Lindström, Tiina Laatikainen, Katja Wikström, Karoliina Nelimarkka, Jemina Kivelä, Päivi Valve, Eeva Virtanen, Greet Cardon, Julie Latomme, Vicky Van Stappen, Nele Huys, Lieven Annemans, Lore Pil, Prof. Dr. Peter Schwarz, Ivonne Panchyrz, Maxi Holland, Patrick Timpel, Konstantinos Makrilakis, Stavros Liatis, George Dafoulas, Christina-Paulina Lambrinou, Angeliki Giannopoulou, Lydia Tsirigoti, Evi Fappa, Costas Anastasiou, Konstantina Zachari, Lala Rabemananjara, Dimitrios Kakoulis, Mayur Mandalia, Maria Stella de Sabata, Niti Pall, Luis Moreno, Fernando Civeira, Gloria Bueno, Pilar De Miguel-Etayo, Esther M Gonzalez-Gil, Maria I Mesana, Germán Vicente-Rodriguez, Gerardo Rodriguez, Lucia Baila-Rueda, Ana Cenarro, Estíbaliz Jarauta, Rocío Mateo-Gallego, Violeta Iotova, Tsvetalina Tankova, Natalia Usheva, Kaloyan Tsochev, Nevena Chakarova, Sonya Galcheva, Rumyana Dimova, Yana Bocheva, Zhaneta Radkova, Vanya Marinova, Imre Rurik, Timea Ungvari, Zoltán Jancsó, Anna Nánási, László Kolozsvári, Remberto Martinez, Marcos Tong, Kaisla Joutsenniemi, Katrina Wendel-Mitoraj.
